# An atlas of the blood virome in healthy individuals

**DOI:** 10.1016/j.virusres.2022.199004

**Published:** 2022-11-17

**Authors:** Bo Feng, Bo Liu, Min Cheng, Jie Dong, Yongfeng Hu, Qi Jin, Fan Yang

**Affiliations:** a NHC Key Laboratory of Systems Biology of Pathogens, Institute of Pathogen Biology, CAMS&PUMC, Beijing 100730, PR China; b China Institute of Veterinary Drug Control, Beijing 100081, PR China

**Keywords:** Virome, Blood, Metatranscriptomic sequencing, Healthy individuals

## Abstract

Emerging evidence indicates that gut virome plays a role in human health and disease, however, much less is known about the viral communities in blood. Here we conducted a direct metatranscriptomic sequencing of virus-like-particles in blood from 1200 healthy individuals, without prior amplification to avoid potential amplification bias and with a strictly bioinformatic and manual check for candidate viral reads to reduce false-positive matches. We identified 55 different viruses from 36 viral families, including 24 human DNA, RNA and retroviruses in 70% of the studied pools. The study showed that anelloviruses are widely distributed and dominate the blood virome in healthy individuals. Human herpesviruses and pegivirus-1 are commonly prevalent in asymptomatic humans. We identified the prevalence of RNA viruses often causing acute infection, like HEV, HPIV, RSV and HCoV-HKU1, revealing of a transmissible risk of asymptomatic infection. Several viruses possible related to transfusion safety were identified, including human Merkel cell polyomavirus, papillomavirus, parvovirus B19 and herpesvirus 8 in addition to HBV. In addition, phages in *Caudovirales* and *Microviridae*, were commonly found in pools of samples with a very low abundance; a few sequences for invertebrate, plant and giant viruses were found in some of individuals; however, the remaining 31 viruses mostly reflect extensive contamination from commercial reagents and the work environments. In conclusion, this study is the first comprehensive investigation of blood virome in healthy individuals by metatranscriptomic sequencing of VLP in China. Further investigation of potential false positives representing a major challenge for the identification of novel viruses in mNGS, will offer a systemic idea and means to reveal true viral infections of human.

## Highlights

• We identified 56 different viruses from 37 viral families, including 25 human DNA, RNA and retroviruses in 70% of the studied pools.
• Anelloviruses are widely distributed and dominate the blood virome in healthy individuals.
• Human herpesviruses and GBV-C are commonly prevalent in asymptomatic humans.
• The prevalence of RNA viruses often causing acute infection, like HEV, HPIV, RSV and HCoV-HKU1, revealing of a transmissible risk of asymptomatic infection.
• Several viruses possible related to transfusion safety were identified, including human Merkel cell polyomavirus, Papillomavirus, Parvovirus B19 and Herpesvirus 8 in addition to HBV and HIV.

## 1 Introduction

The human virome is the repertoire of all viruses found in and on the human body, including eukaryotic viruses, prokaryotic viruses and even endogenous viral elements integrated into host chromosomes (Virgin, 2014; Carroll et al., 2018). So far, the gut virome is the most-studied site in asymptomatic individuals because of an essential role in maintaining gut microbiome structure/function and thereby contributes significantly to human health (Garmaeva et al., 2019; Liang et al., 2020; Manrique et al., 2016). It was shown that a core and common bacteriophage communities are globally distributed and comprise the healthy gut phageome (Manrique et al., 2016). The colonization of the infant gut is first mainly by temperate bacteriophages, and later by eukaryotic viruses; bacteriophages (*Myoviridae, Podoviridae, Siphoviridae* and *Microviridae*) represent a much larger proportion of human gut virome than eukaryotic virus (Liang et al., 2020). A very low abundance of eukaryotic virus is typical of gut virome and the anelloviruses are the most common eukaryotic viruses found in the gut from healthy individuals (Manrique et al., 2016; Liang and Bushman, 2021).

Understanding the blood virome, undoubtedly, is important not only for the transfusion safety, but also for the identification of novel viruses potentially infecting humans. Blood viruses, which can readily migrate via blood throughout the body, are not only crucial for the spread of viruses to almost all tissues and organs following primary infection but are central to the entire strategy for their persistence in the body (Rascovan et al., 2016). However, blood virome is understudied and much less is known about the effects of immune modulation and antiviral therapies on virome composition, though many researches about blood virome were reported as reviewed previously (Rascovan et al., 2016). As we known, most of available studies on blood virome were based on analysis of human genome or transcriptome datasets in database, which is a by-product of these sequencing (Kumata et al., 2020; Moustafa et al., 2017). A few of studies found sequences of many eukaryotic viruses, including anelloviruses that have been proposed as biomarkers of immunocompetence in transplant recipients and HIV patients (Kapoor et al., 2015; De Vlaminck et al., 2013; Cebria-Mendoza et al., 2021). A metagenomic study on RNA viruses in the blood in healthy individuals led to the discovery of two novel rhabdoviruses (Stremlau et al., 2015). Other viral sequences found in the blood of asymptomatic individuals are related to members of the families *Herpesviridae, Picornaviridae, Poxviridae, Flaviviridae, Mimiviridae* and *Phycodnaviridae* (Rascovan et al., 2016). Phage DNA sequences were also found in blood in studies analyzing virus-like particles and circulating DNA, including *Myoviridae, Siphoviridae* and *Microviridae* (Garmaeva et al., 2019).

Metagenomic next-generation sequencing (mNGS) have revolutionized how we think about viruses. We can not only focus on pathogenic viruses (eg. emerging viruses), but also go beyond to the human virome in physiologically healthy individuals and their interactions with each other, with other microbes, and with host genetics and immune systems, and how they affect health and disease (Virgin, 2014). In this work, we explored the human virome in plasma and PBMC from about 1200 healthy individuals in China, none of whom were ascertained for any infectious disease. We aim at comprehensively deciphering the blood virome in Chinese individuals and emphasizing the importance of blood-borne virome in transfusion medicine and the infectious disease.

## 2 Materials and methods

### 2.1 Study population and blood collection

All blood collection was performed by technicians at the Beijing Red Cross blood Center (Beijing, China) during November 2018 to November 2019. Two milliliters of blood were collected from healthy blood donors into sodium heparin vacutainers (BD Biosciences, Franklin Lakes, NJ, USA) and stored at 4°C until processing within 24 hours after blood collection. Written informed consent was obtained from all individuals. The study was approved by the ethics committees of the Beijing Red Cross blood Center.

### 2.2 Purification of VLPs from PBMC and plasma and pooling

**Fig. 1:**
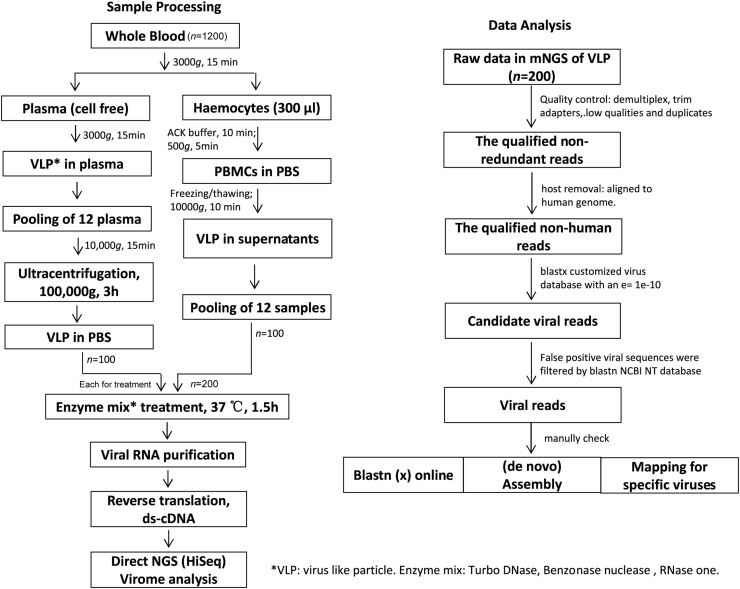
Study design, sample processing and data analysis. The flowchart summarizes the steps followed to enrich virus-like particles (VLP) in PBMC and Plasma, and bioinformatic analysis for viral sequences.

We have combined a series of enrichment methods including filtration and ultra-centrifugation to enrich for virus-like-particles (VLPs) in PBMC and plasma in parallel. Two milliliters of heparin-treated whole blood samples were first centrifuged at 3,000 × g for 10 min at 4°C within 3 hours of sample collection to isolate cell-free plasma and haemocytes (Peripheral Blood Mononuclear Cell, PBMC). Plasma was transferred to microcentrifuge tubes and mixed in a pool of 12, which was centrifuged at 10,000 × g for 10 min to remove residual cells and further ultracentrifuged at 100,000 × g for 3 hours to concentrate the VLPs in 280μl phosphate buffered saline (PBS). In parallel, the cells in precipitation from each plasma-corresponding blood were subjected to Red Blood Cell Lysis Using ACK Lysing Buffer (Thermo fisher), and PBMCs were collected by centrifugation at 300 x g for 5 minutes and resuspended in 150μl of PBS twice. The clean PBMCs were frozen and thawed twice to release viruses cells into supernatant, then centrifuged at 10,000 × g for 10 min and the supernatant of 12 samples were pooled and subjected to further processing (Fig. 1).

### 2.3 Extraction of viral nucleic acids and double-stranded cDNA synthesis

A total of 200 pools (100 pools of plasma, 100 pools of PBMC) were constructed from 1200 healthy donors. The concentrate of VLPs from each pool was treated with a cocktail of Turbo DNase (Ambion), RNase (Promega) and Benzonase (Ambion) at 37 °C for 90 min to degrade nonencapsulated nucleic acids. Finally, each of 200 μl VLP preparation was used for viral nucleic acid extraction using the QIAamp MinElute Virus Spin Kit (Qiagen) immediately and subjected to double-stranded cDNA (ds-cDNA) synthesis SuperScript™ Double-Stranded cDNA Synthesis Kit (ThermoFisher).

### 2.4 Library construction and sequencing

Ds-cDNA was used directly for the construction of the shotgun libraries. The DNA concentration was measured using the Qubit (Invitrogen). Libraries were made using an Illumina *Nextera* XT Samples Prep kit (Illumina). Barcoded libraries were pooled for sequencing. The concentration of the pooled libraries was measured using Qubit (Invitrogen) and the size distribution of the pooled libraries was checked by Agilent Technology 2100 Bioanalyzer using a High Sensitivity DNA chip (Agilent). Sequences were acquired using the HiSeq 2000 (100-bp single-end reads, Illumina). Metatranscriptomic sequencing data is deposited at the Genome Sequence Archive in the BIG Data Center, Beijing Institute of Genomics, Chinese Academy of Sciences, under BioProject accession no. PRJCA008687.

### 2.5 Bioinformatic analysis

Quality control for the blood VLP reads was performed using the in-house bioinformatics pipeline (Fig. 1). In brief, low-quality reads and adaptor sequences were removed by Trimmomatic, low-complexity reads were identified and discarded by Komplexity (https://github.com/eclarke/komplexity) and then duplicate identical sequences were filtered out by BBmap (https://jgi.doe.gov/data-and-tools/bbtools/). Dereplicated reads were aligned using BWA to the host (GRCh38 for human genome) and removed. The quality-controlled unmapped reads were classified by blastx with e-value 1e-10 using a custom database that included all complete human, bacterial, archeal and viral genomes in RefSeq release (released on 9 July 2021). Viral hits were filtered for bit-score ≥ 30. Reads with hits other than viruses with bit scores greater than or equal to the viral hits were discarded. Finally, to reduce false-positive matches, candidate reads with viral hits of the human viruses were manually and visually verified by searching (blastn or blastx) against NCBI nt or nr (online) and by aligning the reads to the corresponding viral genomes. For viruses where only a few reads could be identified, we checked them manually for unambiguous mapping; for viruses with enough reads or present in numerous individuals across many sample, we tried to reconstruct genomes and the reference genome was selected based on BLAST results. The inspection of sequence identity against plasmids and vectors like phiX174 and M13, or against laboratory strain like HCV and ZIKA in use at our laboratory will be regarded as contaminants and removed from downstream analysis . Furthermore, we observed cross-contamination from the viruses with high abundant sequences to other samples on the same flow cell, where the clusters of samples were removed from analysis.

## 3 Results

### 3.1 Data Summary

We performed direct metatranscriptomic sequencing of VLP in 200 plasma pools and PBMC pools (plasma, 100; PBMC, 100) from 1200 healthy individuals in China. Total nucleic acid was extracted from VLP to comprehensively detect both DNA and RNA viruses, and subjected to dsDNA synthesis and further deep sequencing without prior random amplification to avoid potential amplification bias. Illumina sequencing generated a total of 218 GB 101 base pair (bp) single-end reads. After quality control, the remaining high quality, unique, nonhuman reads were then translated and analyzed via protein similarity search (BLASTx) against a comprehensive database of viruses, bacteria, archaea, human, vectors and other eukaryotes. Finally, a total of 5,264,459 nonredundant viral reads [ PBMC: 1,473,239, 14,732 ± 14,972 (total, mean ± sd) per pool; Plasma: 2,592,139, 25,921 ± 30,250 per pool] were obtained (Supplementary Table 1). Under an empirical cut-off of read number ≥ 2, we detected a total of 36 viral families-derived sequences that were presented in at least one pool, including sequences from 24 human DNA viruses, RNA viruses and retroviruses in 70% of the study pools.

### 3.2 Human blood virome

#### 3.2.1 Human DNA viruses in blood

**Fig. 2:**
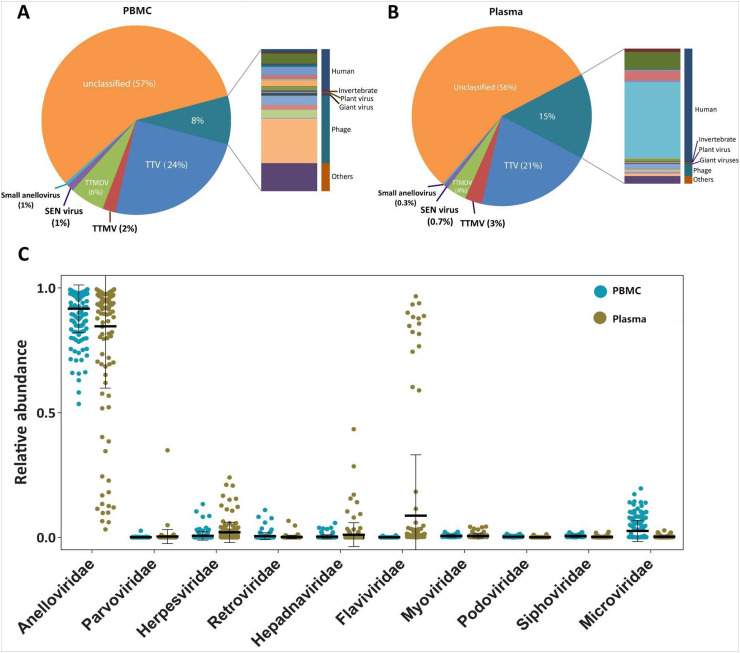
Average relative abundance of viral sequences in the pools of (A) PBMC and (B) plasma. (C) Average relative taxon abundance comparisons between the PBMC and plasma pools at the family level. Median relative abundances were marked as lines and whiskers indicating the relative abundances.

**Anelloviruses dominate the blood virome in healthy individuals.** Anelloviruses were detected in each of the pool, and constituted the most of viral communities (average: 92% in PBMC; 85% in plasma). The *Anelloviridae* fraction was composed mostly of viruses from alphatorquevirus (Torque Teno Viruses, TTVs), betatorquevirus (Torque Teno mini Viruses, TTMVs) and unclassified anelloviruses. In addition, gammatorquevirus (Torque Teno midi Viruses, TTMDVs), SEN viruses and small anelloviruses were also identified (Fig. 2A). These together indicate a great degree of genetic diversity within this group, including high inter- and intra-individual variability. More than 55% of sequences assigned to family *Anelloviridae* were unclassified, indicating the possible presence of new or unknown viruses.

In addition to a significant presence of anelloviruses*,* we identified a few sequences for other human DNA and RNA viruses, phages, invertebrate viruses, plant viruses and giant viruses, which together accounted for 8% and 15% of total viral sequences in PBMC pools and plasma pools, respectively (Fig. 2A and B). At family level, PBMC pools were enriched with *Anelloviridae* and *Microviridae,* while plasma pools were enriched with *Flaviviridae, Herpesviridae, Hepadnaviridae* and *Myoviridae* (Fig. 2C).

#### 3.2.2 Other human DNA viruses in blood

**Fig. 3:**
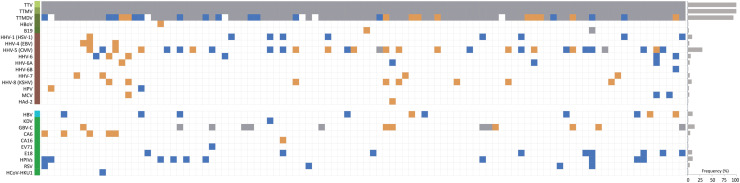
Landscape of the healthy human blood virome. The heatmap shows the presence of reads of specific viruses in PBMC and plasma pools from human blood. The colored boxes indicate whether the viruses were identified in the PBMC or plasma pools, or in both of them. Blue, PBMC pools; Orange: plasma pools; grey, both. The overall frequency of each virus in human blood is summarized in bar graphs at right. The colored column at left denotes viruses with different genome, including ss (+) DNA, ds (+) DNA, retroviruses and ss (+) RNA.

Among the human herpesviruses (HHV), HSV-1 was identified in 8 (8%), CMV was identified in 16 (16%), HHV6A was identified in 3 (3%) and HHV6B was identified in 1 (1%) of the PBMC pools. A few of sequences of HHV6 was also found in 4 (4%) of the PBMC pools, but can't be subtyped to HHV6A or HHV6B. More types of the HHV were detected in plasma pools. HSV-1 was identified in 1 (1%), EBV identified in 3 (3%), CMV was found in 16 (16%), HHV-6 was found in 2 (2%), HHV6A was identified in 1 (1%), HHV-7 was found in 4 (4%), and HHV-8 (KSHV) was found in 8 (8%) of the pools (Table 1, Fig. 3). We identified 4 reads of human papillomavirus in 1 PBMC pool, and 2 reads in 1 plasma pool. Most of sequences for family *Papillomaviridae* cannot be aligned to specific viral species, and regarded as possible unknown viruses. A few of reads of human Merkel cell polyomavirus (MCPyV) were identified in 2 PBMC pools and 1 plasma pool by manual check. This virus was reported to be highly seroprevalent and positive by PCR, but not as in this study. We found a few of reads for human mastadenovirus C in both plasma and PBMC pools, and most of which were confirmed as synthetic construct clone Parva T-Ag, and only 1 plasma pool was confirmed to be positive for human adenovirus 2 with 3 reads. (Table 1, Fig. 3). Human bocavirus was also found in 1 plasma pool with 10 reads matching to VP1, VP2 and NS1 proteins. Human parvovirus B19 (B19), which can spread through blood or blood products, was identified in 1 pool of PBMC with 444, and in 2 pools of plasma, one of which carried 5552 reads assembled into nearly complete genome (Table 1, Fig. 3).

**Table 1:** Detected viruses in PBMC pools (*n* = 100) and Plasma pools (*n* = 100) from 1200 healthy individuals in China.

Viruses	PBMCs pools (*n* = 100)		Plasma pools (*n* = 100)		Overall prevalence
	Prevalence (%)	Reads number (mean)	Prevalence (%)	Reads number (mean)	
Alphatorquevirus (TTV)	100 (100%)	18-3422 (433)	100 (100%)	15-1364 (317)	100
Betatorquevirus (TTMV)	100 (100%)	18-2851 (425)	100 (100%)	26-3980 (959)	100
Gammatorquevirus (TTMDV)	83 (83%)	2-115 (16)	78 (78%)	2-43 (11)	94
unclassified *Anelloviridae*	100 (100%)	321-40906 (5394)	100 (100%)	246-21974 (5465)	100
Human bocavirus (HBoV)	-	-	1 (1%)	10	1
Human parvovirus B19 (B19)	1 (1%)	444	2 (2%)	16- 5552 (2784)	2
Human herpesvirus 1 (HHV-1, HSV-1)	8 (8%)	2-13 (5)	1 (1%)	10	9
Human herpesvirus 4 (HHV-4, EBV)	-	-	3 (3%)	12-23(19)	3
Human herpesvirus 5 (HHV-5, CMV)	16 (16%)	5-2499 (382)	16 (16%)	5-141 (33)	30
Human herpesvirus 6 (HHV-6)	4 (4%)	2-5 (3)	2 (2%)	2-3 (3)	6
Human herpesvirus 6A (HHV-6A)	3 (3%)	2-26 (11)	1 (1%)	2	4
Human herpesvirus 6B (HHV-6B)	1 (1%)	12	-	-	1
Human herpesvirus 7 (HHV-7)	-	-	4 (4%)	2-4 (3)	4
Human herpesvirus 8 (KSHV)	-	-	8 (8%)	3-11 (7)	8
Human papillomavirus (HPV)	1 (1%)	4	1 (1%)	2	2
Merkel cell polyomavirus (MCPyV)	2 (2%)	2-3 (3)	1 (1%)	2	3
Human adenovirus 2 (HAd-2)	-	-	1 (1%)	3	1
Hepatitis B virus (HBV)	7 (7%)	175-667 (312)	3 (3%)	44-2125 (948)	10
Kadipiro virus (KDV)	1 (1%)	33	-	-	1
Human Pegivirus 1 (HPgV-1, GBV-C)	8 (8%)	10-42 (19)	14 (14%)	101-142824 (55441)	14
Coxsackievirus A6 (CA6)	-	-	5 (5%)	3-4 (3)	5
Coxsackievirus A16 (CA16)	-	-	1 (1%)	679	1
Enterovirus 71 (EV71)	1 (1%)	7	-	-	1
Echo virus 18 (E18)	9 (9%)	2-234 (53)	-	-	9
Human parainfluenza viruses (HPIVs)	10 (10%)	4-108 (27)	-	-	10
Respiratory syncytial virus (RSV)	4 (4%)	2-7 (4)	-	-	4
Human coronavirus HKU1 (HCoV-HKU1)	1 (1%)	2	-	-	1

#### 3.2.3 Human RNA viruses in blood

**Fig. 4:**
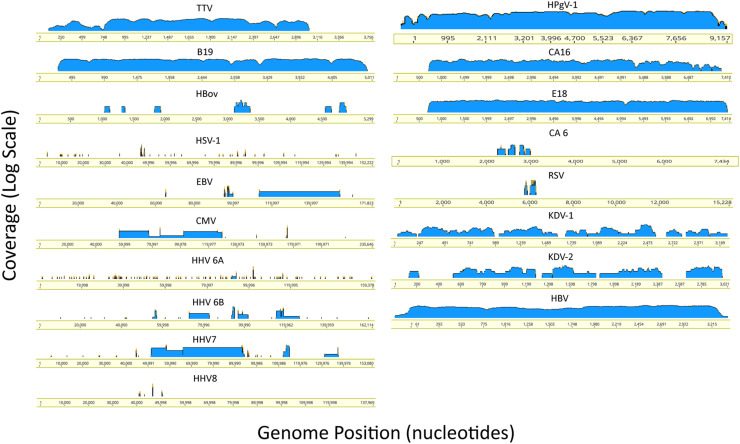
Genome coverage of selected human viruses. Shown are the alignment of reads contributed by all individuals carrying the corresponding virus. The depth of coverage (y-axis) changes in scale as a reflection of the viral abundance and prevalence.

We identified 9 human RNA viruses including human pegivirus-1 (HPgV-1 or GBV-C), parainfluenza virus (HPIV), respiratory syncytial virus (RSV), human coronavirus HKU1 (HCoV-HKU1), enteroviruses (CA6, CA16, EV71 and echovirus 18) and Kadipiro virus (KDV). The blood-borne HPgV-1 was identified and abundant in 14% of plasma pools with a median of 41,501 reads per pool, while in 8 respective PBMC pools of the 14 plasma pools with a median of 19 reads per pools. Upon validation, we identified a cluster of pools with the HPgV-1 in the same flow cell. The sample with the highest viral load led to contamination of samples sharing the same flow cell, which, although positive, were thus classified as contaminants (Table 1, Fig. 3). CA16 was identified in 1 plasma pool with 679 reads, and CA6 was identified in 5 plasma pools with a median of 3 reads. EV71 with 7 reads was identified in 1%, E18 with a median of 53 reads was identified in 9% of the PBMC pools. We also identified a few reads for HPIV in 10%, RSV in 4% and HCoV-HKU1 in 1 (1%) of the PBMC pools. These viruses are commonly associated with acute human infection, we identified them revealing of presymptomatic states or transmissible risk of asymptomatic infection. We also observed Kadipiro-like virus in 5 PBMC pools with a median of 8 reads, showing ∼ 88% aa identities to segments of Kadipiro virus isolated from mosquitoes in China. Kadipiro virus was identified in 5 PBMC pools, however, four positive samples shared the flow cell with the sample with the highest load of viral copies (33 reads) and were classified as cross-contaminants (Table 1 and Supplementary Table 1). In addition, of relevance to transfusion medicine, we identified a few sequences for HBV in 7 PBMC pools and 3 plasma pools(Table 1, Fig. 3). Further, we tried to reconstruct genomes for viruses with enough reads or present in numerous individuals across many samples in order to provide proof of the viral presence confirmed by broad and average coverage of each viral genome (Fig. 4).

We also identified a few sequences for HIV-1 in 2 PBMC pools, and observed the presence of more reads from *Retroviridae* with best match to spleen focus-forming virus. The source of these viruses is likely to be through contamination of cell reagents or the environment. We identified a few of sequences for *Circoviridae*, most of which, however, cannot be aligned to specific viral species, reflecting contamination occurring during experiment process as reported as previously. A few of reads of influenza A virus (H7N9), HCV and Zika virus were also found. However, the inspection of sequences showed 100% identities against our laboratory strains in use, thus they were identified as synthetic vectors and removed from analysis. A few of sequences for *Arenaviridae* and *Astroviridae* were found, however, these were identified as contaminants from environmental contaminants sharing between the flow cell and removed from subsequent analysis. (Supplementary Table 1).

### 3.3 Bacteriophages was observed widely with very low relative abundance

We observed a widespread presence of phage DNA in both of plasma and PBMC pools, mainly derived from the order *Caudovirales* (*Myoviridae, Siphoviridae,* and *Podoviridae*) and family *Microviridae*. At species level, we found 3 shared bacteriophages, including pseudomonas virus EL, tetrasphaera virus TJE1 and bdellovibrio phage phi1422 in more than 50% of healthy individuals. Taxonomic assignment of these viral reads revealed a prevalence of unknown phages belonging to the order *Caudovirales* and the family *Microviridae,* indicating that the majority of bacteriophages are unknown, with only a limited subset that can be taxonomically classified. As described previously, although there is a possibility that some phage DNA could translocate from the gut, the presence of phages always is revealing of contamination. Phage DNA can also derive from bacteria contaminating the reagents. Pseudomonas virus PRD1 in *Tectiviridae* was identified in 34 (29.3%) of PBMC pools, and 29 (20.4%) of plasma pools. We identified a few of sequences of a linear ssRNA phage, acinetobacter phage AP205, in 23 plasma pools with a median of 2 reads per sample, also reflecting environmental contamination (Supplementary Table 1).

A archaeal virus, acidianus bottle-shaped virus, was identified in 8 PBMC and plasma pools with a median of 14 reads per sample. A fungal virus, penicillium chrysogenum virus, was also identified in 6 plasma pools with a median of 2 reads per sample. it was removed because there is a possibility of cross contamination or from baculovirus vectors. Pseudomonas virus PRD1 was also removed because of the suspicion of misshit of transcripts. Notably, escherichia virus phiX174 was only found in 3 pools with 3 reads and removed from our analysis. A few of reads from *Inoviridae* was found as escherichia virus M13 (vector) and removed from our analysis (Supplementary Table 1).

### 3.4 Giant, invertebrate and plant viruses

We identified a few viral sequences of giant virus, pacmanvirus S19 in 4 PBMC pools with a median of 2 reads per pool, and in 3 plasma pools with a median of 3 reads per pool. However, it was difficult to confirm whether it was truly present or the reagent or laboratory contamination. We also found a few of reads distantly *(*aa. Identities, _∼_57%*)* matched to orf virus by blastx in 2 plasma pools with a median of 3 reads per pool, and in 2 PBMC pools with a median of 2 reads per pool. However, after manual inspection, these reads can be aligned to cattle genome by blastn with higher nucleotide similarity (nt. Identities, 95%) and represented false positive. We also identified a few reads of invertebrate viruses (*Iridoviridae, Dicistroviridae* and *Iflaviridae*), and plant viruses (*Rhabdoviridae, Nanoviridae,Geminiviridae, Virgaviridae, Betaflexiviridae, Tombusviridae, Tymoviridae,* and *Phycodnaviridae*). The source of these viruses is likely to be through contamination of reagents or the environment as well as transients from foods. We found a few of reads for *Dicistroviridae* in 52 plasma pools with a median of 2 reads per pool. Although detection of dicistrovirus RNA in blood of febrile Tanzanian children was reported (Cordey et al., 2019), all reads detected here were matched to insect viruses, like aparavirus (aa. Identities, _∼_95%), aphid lethal paralysis virus (aa. Identities, _∼_100%) and other *dicistroviruses* with a distance relative (aa. Identities, _∼_65%), reflecting contamination from reagents or work environment (Supplementary Table 1).

### 3.5 Altered blood virome in individuals with elevated ALT levels

**Fig. 5:**
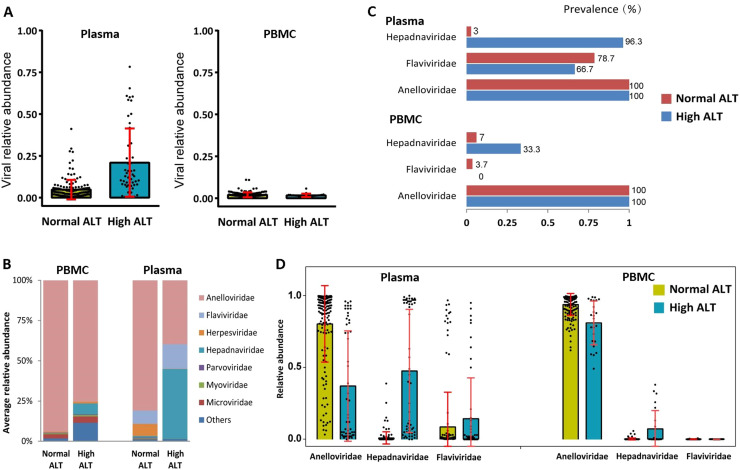
Altered blood virome in individuals with elevated ALT levels in PBMC and Plasma pools. (A) Comparison of viral average relative abundance of total reads between individuals with normal and high ALT levels in PBMC and Plasma pools. (B) Comparison of prevalence of the three most abundant viral families (*Anelloviridae, Flaviviridae* and *Hepadnaviridae*) between individuals with normal and high ALT levels in PBMC and Plasma pools. (C) Comparison of average relative abundance of the most abundant viral families between individuals with normal and high ALT levels in PBMC and Plasma pools. (4) Comparison of relative abundance of the three most abundant viral families (*Anelloviridae, Flaviviridae* and *Hepadnaviridae*) between individuals with normal and high ALT levels in PBMC and Plasma pools.

Alanine aminotransferase (ALT) is very sensitive to liver inflammation, and an ALT test is currently demanded for blood donation to ensure transfusion safety (Li et al., 2019). Herein, we collected 54 blood with high ALT values and conducted a comparison of virome in the blood with or without elevated plasma ALT levels. The relative abundance of viral fraction in plasma with high ALT -was higher than that in normal donors, but not in PBMC (Fig. 5A). Eukaryotic virus, including *Anelloviridae, Hepadnavirida* and *Flaviviridae* together accounted for most of viral sequences in blood with high ALT (Fig. 5B). In high-ALT level plasma, the prevalence and the relative abundance of *Hepadnaviridae* (HBV) were significantly higher than those in normal-ALT plasma (Fig. 5C, 5D).

## 4 Discussion

This study, as we know, is the first study on blood virome in China by direct meta-transcriptomic sequencing of VLP in PBMCs and plasma. Total nucleic acid was extracted from enriched VLP to deeply detect circulating DNA and RNA viruses without being abundant in blood. The libraries were constructed directly from ds-cDNA without prior amplification to avoid potential amplification bias and contamination.

We found 55 different viruses from 36 viral families, including 24 human DNA, RNA and retroviruses, however, the remaining 31 viruses of them represented false positive or contamination and a massive effort was required to rule out them. For example, we found HIV-1 in one pool of PBMC with 65 reads, all of which were mapped to synthetic HIV-1 clone pSF256.8 upon further inspection. And a few reads for Zika virus were found in 5 PBMC pools and 2 plasma pools, however, all of them were mapped to a synthetic construct sequence. Thus, they were removed from subsequent analysis. Phage DNA could translocate from the gut, and can also derive from bacteria contaminating the reagents (Moustafa et al., 2017; Gorski et al., 2006). However, it is difficult to distinguish them from contaminants. We observed a widespread presence and very low abundance of phage DNA similar to those of gut virome (Liang et al., 2020; Koonin et al., 2021), and these may reflect environmental contamination. In the study, we didn't observe a very significant presence of phage phiX174 DNA that was used as commercial materials (Moustafa et al., 2017). We also found several possible contamination situations: The presence of samples with high viral-titers leads to cross-talk among samples and misidentification of samples described as “sample bleeding” that refers to the incorrect assignment of reads to multiplexed samples that are being sequenced in the same sequencing lane as described previously (Moustafa et al., 2017). In the study, flow cells with high-titer HPgV-1 samples contained other positive samples that were regarded as potential false positives. The infections of H7N9 and HCV were identified with 100% sequence similarity to the reference viral sequences that was constructed in our laboratory. Therefore, we excluded these viral infections to avoid counting false positives.

The study found that anelloviruses were dominant in viral communities and widely distributed among healthy individuals. However, more than 55% of sequences assigned to this family were unclassified indicating a lot of novel or unknown viruses remained to be studied as indicated previously. In addition, we identified 23 viruses associated with human infection in 70% of the study pools, which included not only the DNA virome, such as 6 different HHV types, HPV, MCPyV, HAdv, parvovirus B19, HBoV, but also the RNA virome such as HPgV-1, KDV, HEV (EV71, CA6, CA16 and E18), HPIVs, RSV and HCoV-HKU1. These viruses generally correspond to those known to be prevalent in the human population. Notably these viral sequences detected in pools of samples may overestimate the prevalence of the specific virus.

We identified 6 types of the herpesviruses with the exception of HSV-2 that was sexually transmitted viral infection affecting the skin or mucous embranes of the genitals (Zhang et al., 2022; Adelson et al., 2005), and varicella-zoster virus (HHV-3), which was commonly detected in blood from immunosuppressed hosts and in immunocompetent subjects with active herpes zoster disease (Kronenberg et al., 2005). It was observed that HHV4, HHV7 and HHV8 were appeared exclusively in plasma, and this difference may be due to different stages or sites of infection with viruses, in addition to specimen types. Although the viral DNA was previously identified via PCR amplification in 8.3% of healthy Australian male blood donors, we observed HPV reads only in 1 pool (<1%) (Chen et al., 2009). MCPyV has been found to be associated with Merkel cell carcinoma and chronic lymphocytic leukemia and reported in 22% of blood samples from healthy donors using PCR (Pancaldi et al., 2011). MCPyV antibodies have been detected in as many as 0.6% of healthy blood donors (Pastrana et al., 2009). In our study, MCPyV was identified in 1% (1/100) of the pools. B19 was detected more frequently in plasma pools than in PBMC pools. B19 and other parvoviruses are of concern to transfusion safety because these viruses are not routinely screened. As expected, we identified the presence of HBV in both of PBMC and plasma pools. The blood samples obtained in the study were qualified after the preliminary screening, and our study thus highlights the necessity for further laboratory testing of the virus by molecular methods. HPgV-1, which is distantly related to hepatitis C virus, was the most prevalent and abundant RNA virus in addition to anelloviruses in this study. HCV positive individuals are often coinfected with HPgV-1 (Samadi et al., 2022; Reshetnyak et al., 2008), however, we didn't find the case. HPgV-1 is highly prevalent and genetically diverse chronic human viral infections with no clinical symptoms. Transmission of the HPgV-1 during transfusion was reported. HEV, HPIVs, HCoV and RSV, which commonly are regarded as causes for human acute infection, were found in healthy individuals or asymptomatic carriers. These viruses may be potentially transmitted via blood products or reflect a potential risk of transmission susceptible individuals like children through asymptomatic infection. Kadipiro virus was isolated from Culex fuscocephalus mosquitoes in 1981, and proposed to genus Seadornavirus in 2000 (Attoui et al., 2000). Since then, KDV has been isolated from mosquitoes in China (Sun et al., 2009, Zhang et al., 2018) and detected in Denmark bat (Lazov et al., 2021). KDV was also detected in a plasma sample from a febrile adult in Kenya in 2016, but the results were regarded as contaminants due to fail in confirmation (Ngoi et al., 2016, Ngoi et al., 2017). Although it is not known whether KDV is able to infect humans or other mammals via vectors as seen with Banna virus, it is insufficient to rule out the possibility as so far.

This study has the following limits. First, we conducted a complete search by Blastx. This approach takes a lot of time and computing power and not suitable for timely testing. However, it serves to identify viral sequences not only prevalent as we know, but also those distantly related to known viruses in database. Second, the pooling of samples was adapted to enrich VLP as much as possible. However, this may overestimate the frequency of the specific viruses. In preliminary test, we realized that no adequate amount of nucleic acid can be obtained without pre-amplification for sequencing in individual sample and we thus adapted the pooling of sample. Third, as implied in previous reports (Moustafa et al., 2017; Kapoor et al., 2015; Hu et al., 2015; Kandathil et al., 2021), relatively low abundant sequences might be attributed to the contamination from commercial reagents and the environment or mistakes in the demultiplexing of NGS reads. In this study, we identified a significant presence of phages and invertebrate viruses, and we thus excluded phages from blood virome, because it was difficult to distinguish whether they are authentic existence or the possibility of cross-contamination from endogenous retroviral sequences.

In conclusion, this study comprehensively defined the virome in Chinese population through direct metatranscriptomic sequencing of VLP circulating in the blood, providing us for basic data and insight into blood microecology and potential blood-borne infection. It is also emphasized that mNGS is a powerful approach for surveying viral infections, however, there are still several challenges for excluding false positives and identifying real viral pathogen.

## CRediT authorship contribution statement

**Bo Feng:** Methodology, Resources, Investigation. **Bo Liu:** Software, Data curation. **Min Cheng:** Visualization, Writing – original draft. **Jie Dong:** Methodology. **Yongfeng Hu:** Conceptualization, Visualization, Writing – original draft, Writing – review & editing. **Qi Jin:** Conceptualization. **Fan Yang:** Conceptualization, Supervision.

## Declaration of Competing Interest

The authors declare that they have no known competing financial interests or personal relationships that could have appeared to influence the work reported in this paper.

## Data Availability

Data will be made available on request.

## Appendix Supplementary materials

Image, application 1
